# *Pandanus odoratissimus* (Kewda): A Review on Ethnopharmacology, Phytochemistry, and Nutritional Aspects

**DOI:** 10.1155/2014/120895

**Published:** 2014-12-22

**Authors:** Prafulla P. Adkar, V. H. Bhaskar

**Affiliations:** ^1^Department of Pharmacology, JSPM's Jayawantrao Sawant College of Pharmacy and Research, University of Pune, Pune, Maharashtra 411028, India; ^2^Vinayaka Missions University, Sankari Main Road, NH-47, Ariyanoor, Salem, Tamil Nadu 636308, India; ^3^Department of Pharmaceutical Medicinal Chemistry, Gahlot Institute of Pharmacy, Plot No. 59, Sector No. 14, Kopar khairane, Navi Mumbai, Maharashtra 400709, India

## Abstract

*Pandanus odoratissimus *Linn. (family: Pandanaceae) is traditionally recommended by the Indian Ayurvedic medicines for treatment of headache, rheumatism, spasm, cold/flu, epilepsy, wounds, boils, scabies, leucoderma, ulcers, colic, hepatitis, smallpox, leprosy, syphilis, and cancer and as a cardiotonic, antioxidant, dysuric, and aphrodisiac. It contains phytochemicals, namely, lignans and isoflavones, coumestrol, alkaloids, steroids, carbohydrates, phenolic compounds, glycosides, proteins, amino acids as well as vitamins and nutrients, and so forth. It is having immense importance in nutrition. A 100 g edible* Pandanus *pericarp is mainly comprised of water and carbohydrates (80 and 17 g, resp.) and protein (1.3 mg), fat (0.7 mg), and fiber (3.5 g).* Pandanus *fruits paste provides 321 kilocalories, protein (2.2 g), calcium (134 mg), phosphorus (108 mg), iron (5.7 mg), thiamin (0.04 mg), vitamin C (5 mg), and beta-carotene (19 to 19,000 *μ*g) (a carotenoid that is a precursor to vitamin A).* Pandanus *fruit is an important source of vitamins C, B_1_, B_2_, B_3_, and so forth, usually prepared as a* Pandanus *floured drink. Traditional claims were scientifically evaluated by the various authors and the phytochemical profile of plant parts was well established. The methods for analytical estimations were developed. However, there is paucity of systematic compilation of scientifically important information about this plant. In the present review we have systematically reviewed and compiled information of pharmacognostic, ethnopharmacology, phytochemistry, pharmacology, nutritional aspects, and analytical methods. This review will enrich knowledge leading the way into the discovery of new therapeutic agents with improved and intriguing pharmacological properties.

## 1. Introduction

**Figure 1:**
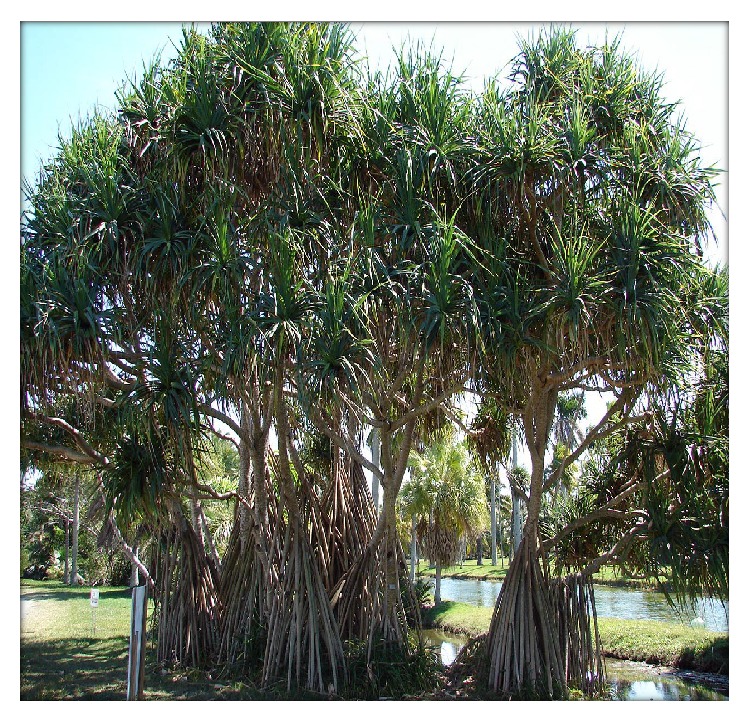
Whole plant;* Pandanus odoratissimus* Linn. (Family: Pandanaceae).

The Indian Ayurvedic plant (kewda)* Pandanus odoratissimus* Lam. belonging to the family Pandanaceae (Figure 1) [1]. The overall* Pandanus* genus contains about 600 species distributed mainly in subtropical and tropical regions; there are around 30 to 40 species of* Pandanus* in India. It is widely distributed in India over coastal districts of Orissa (especially in Ganjam), Andhra Pradesh, Tamil Nadu, and to some extent in parts of Uttar Pradesh [2].* P. odoratissimus* is said to be a restore health, strength, or well-being, promoting a feeling of well-being in tropical climates. Ayurvedic science has found the medicinal action of essential oil yielded by the screw pine's highly scented flowers to be useful in headaches, earaches and as a liniment for rheumatic pains. It may be chewed as a breath sweetener or used as a preservative in rice made foods.* Pandanus* has antiviral, antiallergy, antiplatelet, anti-inflammatory, antioxidant, and anticancer action [3, 4].* P. odoratissimus* naturally occurs in high water marking the very edge of the sea and near coastal forests in Southeast Asia, including the Philippines and Indonesia, extending eastward through Papua New Guinea and northern Australia, and throughout the pacific ocean beaches, including Melanesia (Solomon Islands, Vanuatu, New Caledonia, and Fiji), Micronesia (Palau, Northern Marianas, Guam, Federated States of Micronesia, Marshall lands, Kiribati, Tuvalu, and Nauru), and Polynesia (Wallis and Futuna, Tokelau, Samoa, American Samoa, Tonga, Niue, Cook Islands, French Polynesia, and Hawaii) [4].

## 2. Historical Perspectives

*P. odoratissimus* Linn. is native to South Asia and India has the tradition of alternative therapies; there are no procedures to test the safety and efficacy of traditional remedies and to standardize their effective cure. For these reasons it is essential to increase our efforts in the area of medicinal plant research and exploit it efficiently for the benefit of humanity.

## 3. Habitat

*P. odoratissimus* Linn. forest habitat [5] and usually elevations of sea level to 20 m (66 ft), but can grow at elevations of 600 m (1970 ft) or higher [6]. Kewda plants are found growing along seashores, banks of rivers, ponds, canals, and so forth [7]. It grows in tropical climate, where it can withstand drought salty spray and strong wind. It propagates readily from seed but it is also propagated from branch cutting for farm or for garden. It grows fairly quickly [8].

*Pandanus* plant is also called “pandan” and it is native to Asia and even tropical parts of Australia.* Pandanus* leaves are mostly used in the Southeast Asian cooking (Thomson et al., 2007).

## 4. Cultivation and Collection

Cultivation of* P. odoratissimus* is too little in India, precisely, the Ganjam district in southern Orissa. The plant can be propagated by off sets or division of the suckers. For raising scented types, a fertile, well-drained-soil is preferable. The tree begins to flower 3 to 4 years after planting. The flowering period is rainy season (July–October). The flowers are harvested early in the morning, and the spa dices take a fortnight to mature, depending upon the weather conditions. In India and Burma, the male flowers are valued for their fragrance and some kewda products. Highly prized by Indian perfumer a fully mature tree bears 30–40 spadices in a year. It is estimated that there are about 30–40 thousand trees in Ganjam district and nearly to a million spa dices are annually used for the production of kewda attar, kewda water, and kewda oil [9].

## 5. Pharmacognosy [17]

See Tables 1 and 2.

## 6. Microscopic Characteristics [17]

### 6.1. Transverse Section of Leaf (Surface View)

Transverse section of leaf showed the presence of single layered upper and lower thin walled epidermal cells, with a moderately thick cuticle, and cells are more or less rectangular. Covering type, unicellular, thick walled, lignified Trichomes, pointed at one end and has a base like that of a hockey stick are emerge from the epidermal layers. Stomata are also seen in the epidermal layer. Mesophyll forms the bulk and is differentiated into thin walled, large, polyhedral, colorless parenchyma with intercellular spaces and 3 to 4 layered, tightly arranged spongy parenchyma (Chlorenchyma). Numerous bundles of acicular rap hides and calcium oxalate crystals as prisms were seen in the parenchymatous cells of mesophyll. Collateral vascular bundles were seen at regular intervals and have protoxylem followed by metaxylem towards upper epidermis and phloem followed by bundle sheath extension (sclerenchyma) towards lower epidermis. The whole vascular bundle is covered by border parenchyma. The TS of the leaf when treated with safranin vascular bundles have stained with pink color and when treated with Sudan red lignified cell wall produced red color.

### 6.2. Organoleptic Characteristics

Coarsely powdered shade dried leaf of* P. odoratissimus* Linn. is light green in color with characteristic odor and acrid taste.

### 6.3. Powder Microscopy

It primarily consists of Scalariform and annular xylem vessels; covering type, unicellular, thick walled Trichomes which are lignified and pointed at one end and has a base like that of a hockey stick; paracytic stomata with straight walled epidermal cells surrounding it. Calcium oxalate crystals as prism and acicular raphids scattered in parenchyma.

### 6.4. Leaf Surface Data

Stomata number and stomata index of leaf of* P. odoratissimus* Linn. were carried out. The value of stomata index of upper epidermis is 23 and lower epidermis is 56.

### 6.5. Growth Response

Stem growth is slow to moderate, 2–80 cm (0.8–31 in) per year. Growth and development vary with sex of plant (male or female), variety, and types of planting stock (seedling or branch cutting). For seedling plants, there is a 4–9-year semiprostrate juvenile phase, followed by an erect trunk growth phase of 5–12 years, and then a sexual/flowering phase of 40 or more years. Male plants are usually more branched, up to about 30 branches (maximum 60), than females, up to about 15 branches (maximum 30). The rate of stem growth varies from very slow to moderate (2–80 cm (0.8–31 in) per year). Branch diameter is usually reduced by 10–30% at each branching, and branching ceases when branch diameter is less than about 3.5 cm (1.4 in) in males and 4.5 cm (1.8 in) in females. The life span of established* Pandanus* plants is typically about 50–80 years (but longevity may be much greater, as long as 100–150 years in some environments). The productive fruiting life of vegetatively propagated plants may be only 20–25 years. Senescence is associated with a gradual decline in branch diameter, leaf size, and number of live branches. Branch death is due to the death of the apical meristem, mainly due to insect damage or breakage. Plants developed from branch cuttings usually grow much faster in earlier years than seedling-derived plants, for example, elongating about 50–80 cm (20–31 in) per year, and branch from a lower height [6].

## 7. Phytochemistry [17]

### 7.1. Phytochemical Extracts

Percentage yield and physical characteristics of various extracts of leaf* Pandanus* are shown in Table 3.

### 7.2. Chemical Constituents

Phytochemical structures in* Pandanus odoratissimus* Linn. are included in Table 4.

The principle constituent is the kewda oil, isolated from the inflorescences of* Pandanus*. The chemical composition of this essential oil, obtained by hydrodistillation of staminate inflorescences of kewda (*P. odoratissimus*), when subjected to high resolution GC (gas chromatography) and GC-MS (gas chromatography and mass spectrometry) has been shown to yield ether (37.7%), terpene-4-ol (18.6%), *α*-terpineol (8.3%) and 2-phenylethyl alcohol (7.5%), benzyl benzoate (11%), viridine (8.8%), and germacrene-B (8.3%) along with a small amount of benzyl salicylate, benzyl acetate, benzyl alcohol, and so forth; ethnobotanically kewda oil is used in earache, headache, arthritis, debility, giddiness, laxative, rheumatism, small pox, and spasms. The methanol and aqueous extracts of the leaves of* Pandanus* were subjected to preliminary phytochemical screening and they were tested for the presence of alkaloids, carbohydrates, proteins, steroids, sterols, phenols, tannins, terpenes, flavonoids, gums and mucilage, saponins, and glycosides [10]. The total phenolic content in the aqueous extract was ranged from 3.5 to 10.8% w/w phenolic which are the largest groups of phytochemicals and have been said to account for most of the antioxidants activity of plant extracts [21, 35]. Physcion, cirsilineol, n-triacontanol, *β*-sitosterol, camphosterol, daucosterol and palmitic acid, and steric acid in rhizomes have been reported [24].

Phytochemicals chemical analysis of the root extracts of* P. odoratissimus* led to the isolation of phenolic compounds, lignans type compounds, and some benzofuran derivative. *α*-terpineol, *β*-carotene, *β*-sitosterol, benzyl-benzoate, pinoresinol, germacrene-B, vitamin C, viridine. tangeterine, 5,8-hydroxy-7 methoxy-flavone, vanidine. Among them, pinoresinol and 3,4-bis(4-hydroxy-3-methoxybenzyl)tetrahydrofuran showed strong antioxidative activities when BHA was used as a standard in the thiocyanate method. The new compounds were identified as 4-hydroxy-3-(2′,3′-dihydroxy-3′-methylbutyl)-benzoic acid methyl ester and 3-hydroxy-2-isopropenyl-dihydro-benzofuran-5-carboxylic acid methyl ester, by spectroscopic analysis.

The methanol extract of* P. odoratissimus* was subjected to column chromatography to isolate a total of 15 compounds. Steroids, including phytosteroid mixtures; a-spin sterol and stigmast-7-en-3b-ol mixture; a-spinasterol caproate; stigmast-4-en-6b-ol-3-one and three phenolic compounds; vanillin (1); 2 (E)-3-(3′-methoxy-4-hydroxyphenyl)-prop-2-enal (2); 4-hydroxy-3-(2′,3′-dihydroxy-3′-methyl-butyl)-benzoic acid methyl ester (3) and a new benzofuran derivative, 3-hydroxy-2-isopropenyl-dihydrobenzofuran-5-carboxylic acid methyl ester (4); plus six lignans; eudesmin (5); kobusin (6); pinoresinol (7); epipinoresinol (8); de-4′-O-methyleudesmin (9); and 3,4-bis(4-hydroxy-3-methoxy-benzyl)-tetrahydrofuran (10), were isolated and identified by comparing their data with authentic materials on the basis of their mass, UV, IR, and 1H and 13C NMR spectra [36]. And total synthesis of four* Pandanus* alkaloid: Pandamarilactonine-A, and B, and their chemical precursors non Pandamarilactonine-A and B [37].

## 8. Nutritional Aspects and Staple Food (Figure 2 and Table 5)

### 8.1. Staple Food

**Figure 2:**
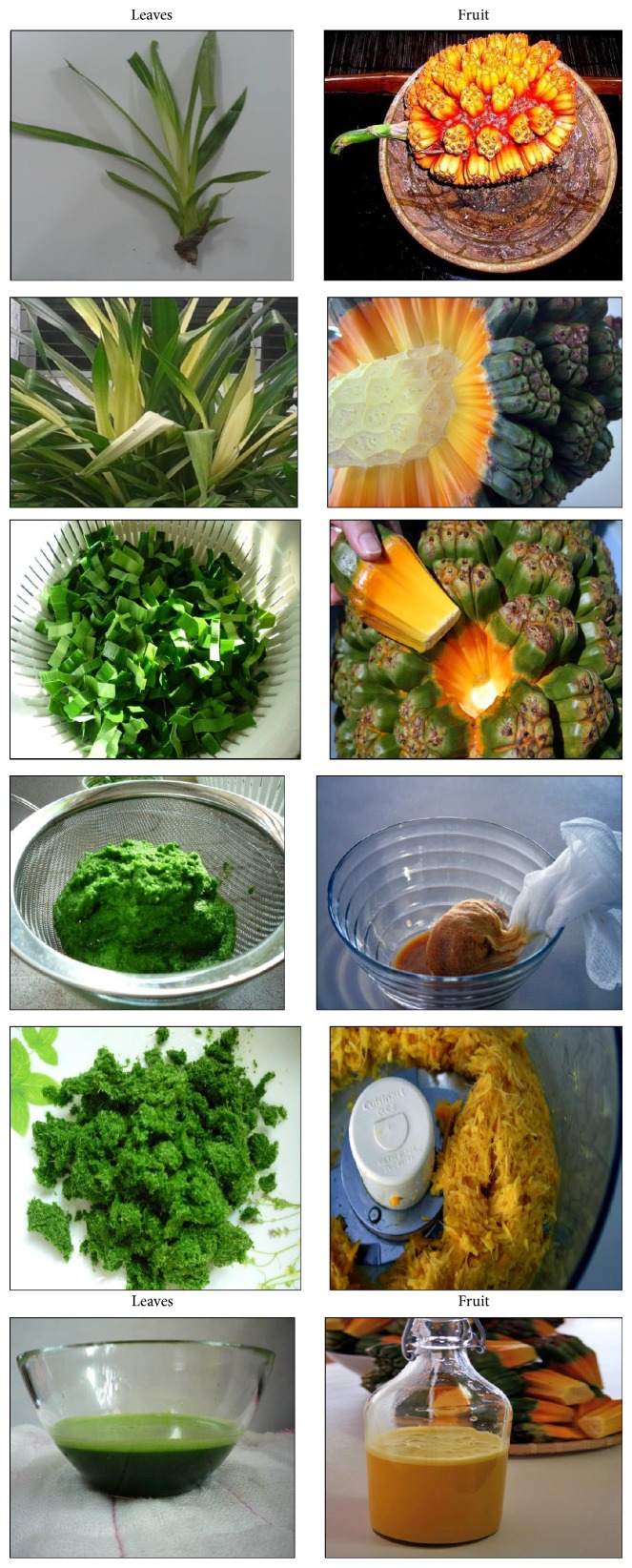
Nutritional aspects and staple food of* Pandanus*.

*Pandanus* fruits are a staple food in parts of Micronesia including the Marshall Islands, Federated States of Micronesia, and Kiribati providing up to 50% of energy intake [11, 15]. They are also widely consumed on Tokelau and Tuvalu [11]. In some places the consumption of* Pandanus* has decreased in recent decades due to the availability of imported foods; for example,* Pandanus* was formerly a major staple food in Nauru [38]. In Micronesia adults may commonly consume 20 fresh keys or about 1 kg (2.2 lb) of fruit per day. The fruit pulp is preserved in several different ways. A paste, which is compared to dates in taste, texture, and appearance, is made by boiling and baking the keys, followed by extracting, processing, and drying the pulp. Cultivars with large amounts of pulp are preferred, and the taste differs among cultivars. On average, 100 g* Pandanus* paste provides 321 kilocalories, 2.2 g protein, 134 mg calcium, 108 mg phosphorus, 5.7 mg iron, 0.04 mg thiamin, 2 mg vitamin C [14–16], and from 390 to 724 *μ*g/100 g beta-carotene (a carotenoid that is a precursor to vitamin A), depending on variety and coloration [12, 13]. Fresh* Pandanus* is an important source of vitamin C. Preserved* Pandanus* pulp mixed with coconut cream makes a tasty, sweet food item.* Pandanus* can also be made into flour that is consumed in different ways, usually prepared as a drink (Figure 2).

### 8.2. Fruit

The keys of selected edible cultivated varieties, those with low amounts of calcium oxalate crystals, are consume draw or cooked. Juice and jam may also be prepared from the fruit. In parts of Micronesia, chewing* Pandanus* keys is usually done outside of meal times and is a pleasurable, highly social activity. Adults may typically consume 20–50 keys daily during the main fruiting seasons [11].

A 100 g portion of edible pericarp is mainly comprised of water (80 g) and carbohydrates (17 g). There are also significant levels of beta-carotene (19 to 19,000 *μ*g) and vitamin C (5 mg), and small amounts of protein (1.3 mg), fat (0.7 mg), and fiber (3.5 g) [11–13]. The edible flesh of deeper yellow- and orange-colored varieties contain higher provitamin A carotenoid levels. The fruit of these varieties has considerable potential for alleviating vitamin A deficiency in Micronesia [11]. As carotenoid-rich food may protect against diabetes, heart disease, and cancer, the consumption of* Pandanus* may also alleviate these serious emerging problems of the Pacific.* Pandanus* fruit is also a useful source of vitamin C (ascorbic acid), thiamine, riboflavin, and niacin (vitamin B-3) [14, 15]. The fruit of wild forms of* Pandanus* contains oxalate crystals that irritate the mouth unless broken down by cooking. The ripe fruit of wild forms may be consumed following cooking and straining the pericarp, but they are not especially palatable or sweet (Table 5).

### 8.3. Nut/Seed

The small seeds of a few varieties of* P. tectorius* are eaten. A similar species,* P. dubius*, has larger seeds that are eaten.

### 8.4. Beverage/Drink/Tea

Juice pressed from the fruits is sweet and slightly acid with a pungent flavor [15]. It is being produced commercially in the Marshall Islands.

## 9. Traditional Uses and Products

Different parts of the* Pandanus* plant are used to provide a myriad of end products throughout the Pacific Islands, especially on atolls. The trunk and large branches are commonly used for building materials in house construction and for ladders. They are also used to make headrests/hard pillows, vases, and fish traps, as sources of glue or caulking for canoes, to extract cream from grated coconuts, and as an aid in making string. Trunks and branches may be burnt for fuel wood or used to make compost. Prop or aerial roots are used in fabrication of house walls and as supports, basket handles, paintbrushes, and skipping ropes. They are also used to produce dyes and in production of traditional medicines. The leaves of selected varieties are treated by soaking in the sea and/or boiling or heating and dying and are then used to make mats, baskets (for ladies and to keep valuables), hats, fans, pillows, canoe sails (formerly), toys, and other plaited wares. The leaves are also used for thatching (both walls and roofs) and for making compost, including special composting baskets woven around the base of giant swamp taro, cigarette wrappers, balls for children's games, and ornaments. They are used for traditional medicines and as a cooking aid in some recipes. The young leaves are used in traditional medicine and for lancing boils, making fans, decoration, and pig feed. Throughout the atoll island countries of the central/northern Pacific, the fleshy keys of the fruits of many traditionally selected, named, and cultivated varieties are consumed fresh or made into various preserved foods. The fruits are also consumed in Solomon Islands and Papua New Guinea. In Polynesia the fragrant, ornamental fruits of different varieties are strung into leis or garlands and used to make perfume. The fibrous, dried, mature drupes are used as paint brushes for painting tapa, for fuel, and for compost, and as fishing line floats. In Kiribati the fruit may also be used as bait for catching lobster. The fragrant male flowers are used to scent coconut oil, perfume tapa cloth, and make garlands.

### 9.1. Animal Fodder

Leaves, particularly young leaves, are recorded as providing fodder for domestic animals such as pigs and horses.

### 9.2. Masticant/Stimulant

Male* Pandanus* flowers have been credited with aphrodisiac properties in Marshall Islands.

### 9.3. Beautiful/Fragrant Flowers

The highly fragrant male flowers are widely used for decoration.

### 9.4. Timber

The stems are used in house construction and also for making ladders, especially on atoll islands. Male trees have hard, solid trunks with a yellow interior containing dark brown fiber bundles. The male wood is very strong, but brittle, meaning that it can suddenly break under a heavy load. It is also a difficult wood to split. Trunks of female trees are hard on the outside, but soft, pithy, or juicy in the interior [39]. Slats made from the clean, dried aerial/prop roots are used for walls of houses and food cupboards.

### 9.5. Fuel Wood

In the northern Pacific, the discarded, dried keys are highly prized as fuel wood for cooking because they are slow burning and therefore preferred for barbecues. The trunk and branches are occasionally used as fuel wood where other fuel wood is scarce, such as on atolls.

### 9.6. Craft Wood/Tools

The wood has many craft uses, such as headrests/pillows, vases, and as an aid for string making and extracting coconut cream. It was formerly used to make weapons (lances and batons). When the flesh is removed from the inner end of a dried key, fibrous bristles are exposed. The bristle end can be used as a brush for decorating tapa, with the hard, woody outer end acting as a handle. Fish traps are made out of the aerial roots in Kiribati.

### 9.7. Canoe/Boat/Raft Making

The trunk of one variety in the Marshall Islands is used to make the masts of traditional canoes. In Hawaii* Pandanus* leaves were the traditionally main material for making canoes ails [40].

### 9.8. Fiber/Weaving/Clothing

In many Pacific countries* Pandanus* leaves are used to weave traditional items of attire, including mats for wearing around the waist in Tonga, as well as hats and various types of baskets.

### 9.9. Rope/Cordage/String

The roots are made into skipping ropes and basket handles. String or cordage is made from the cleaned and dried prop roots.

### 9.10. Wrapping/Parcelization

The leaves are used to wrap tobacco/cigarettes in Micronesia.

### 9.11. Thatch/Roofing/Mats

*Pandanus* leaves are used to weave traditional floor mats in many Pacific countries, as well as in the construction of traditional houses (thatch for walls and roofing). A roof made from* Pandanus* leaves is said to last about 15 years, while one of coconut leaves may last only 3 years [39].

### 9.12. Resin/Gum/Glue/Latex

The trunk is a source of glue or caulking for canoes.

### 9.13. Body Ornamentation/Garlands

Leaves, often neatly cut, fragrant fruits, and flowers are used in making garlands or leis.

### 9.14. Ceremonial/Religious Importance

*Pandanus* is sometimes considered to have supernatural and magical properties in parts of Micronesia and Hawaii. In Kiribati it may be used as a ceremonial food, while in Indonesia the male flowers are used in ceremonies.

### 9.15. Other Uses

In Kiribati and the Marshall Islands the leaves are formed into a ball for use in a kicking game. The trunks of female trees are hard on the outside but soft or juicy in the interior. The female trunks have been used as water pipes after removing the soft interior [39].

In the Philippines, pandan* leaves* are being cooked along with rice to incorporate the flavor and smell to it. As can be observed, the uses of the pandan tree are not limited to cooking uses. Its leaves and* roots* are found to have medicinal benefits. Such parts of the plant have been found to have essential oils, tannin, alkaloids, and glycosides, which are the reasons for the effective treatment of various health concerns. It functions as a pain reliever, mostly for headaches and pain caused by arthritis, and even hangover. It can also be used as antiseptic and antibacterial, which makes it ideal for healing wounds.

In the same manner, a preparation derived from the* bark* of this plant may be used to address skin problems. Many people have also discovered that it is an effective remedy for cough. In India, pandan leaves are being used to treat skin disorders like leprosy and smallpox. The bitter tasting quality of the leaves makes it ideal for health problems which include, but are not limited to, diabetes fever, ulcer, and wounds.

In Hawaii, pandan* flowers* are being chewed by mothers who later give the chewed flowers to their children, as laxative. The juice extracted from pounded roots of this tree are issued and mixed with other ingredients to ease chest pains. Also, it is used as tonic for women who have just given birth and who are still in weak states. Pandan flowers have also been traced with characteristics that function as aphrodisiac. Pandan also manifests anticancer activities, and that is why modern researches in the United States have subjected this plant for further experiments and investigation [41].

Sometimes it is oven dried and kept in bottles for preservation until it is used. This product is mostly available in western countries in dried form. Other than that there are many recipes. To impart its aroma into chicken, pieces of marinated chicken are enclosed in a clever wrapping of “*Pandanus*” leaves and grilled or deep fried. The leaves which are pounded and strained (or blended with a little water) impart flavor and color into cakes and sweets. This flavor is a delicacy to Asians and is as important as vanilla to Westerners. These leaves are used to make small containers for sweets, jelly, and puddings. Some people wrap their hot foods using these leaves as they produce aroma when the food is still warm. These plants can be easily propagated by side shoot cuttings taken from the base of the plant. The higher the maturity of the cutting is, the easier the establishment is [41].

Supercritical carbon dioxide (SC-CO2) and Soxhlet extraction using hexane as solvent were used to extract 2-acetyl-1-pyrroline (2-AP) from pandan leaves. The effect of different extraction pretreatments such as particle size and drying on the extraction yield and concentration of 2-AP were investigated. The identification and quantification of 2-AP were carried out by gas chromatography-mass spectrometry and gas chromatography-flame ionization detector, respectively. This work aims to provide an understanding of the phenomena that occur during cooking and storage, typically on the changes of 2-AP absorption when cooking rice grains with pandan leaves. The parameters investigated were cooking method of excess and optimal water conditions. Even though low in yield and the fact that the 2-AP concentration was obtained from supercritical carbon dioxide extraction, the extracts were pure without any contamination. The grinding and freeze-drying method revealed the best pretreatments for supercritical extraction. The absorption of 2-AP during the cooking of rice grains did not smoothly increase with time. This unexpected result indicated that the phenomena occurring during cooking are quite complex. This work also quantified the potential of pandan leaves to enhance the flavour of cooked rice, particularly under excess water conditions. Storage for 15 min at 24.0 ± 1.0°C is considered as the optimum time for obtaining cooked rice with a high quality of flavor [42].

## 10. Ethnomedicinal Uses

*Pandanus* is a very important medicinal plant, with certain varieties sometimes preferred for particular treatments. Leaves, especially the basal white section of young leaves, and roots are used. In Kiribati,* Pandanus* leaves are used in treatments for cold/flu, hepatitis, dysuria, asthma, boils, and cancer, while the roots are used in a decoction to treat hemorrhoids. In Hawaii the main parts used in making traditional medicines are the fruits, male flowers, and aerial roots [40]. These are used individually or in combination with other ingredients to treat a wide range of illnesses, including digestive and respiratory disorders. The root is used in Palauto to make a drink that alleviates stomach cramps, and the leaves are used to alleviate vomiting [43]. The root is also known for its use in traditional medicine in Pohnpei for STD'S, namely, syphilis [17, 18, 44] (Table 6).

## 11. Pharmacology

A summary of reported pharmacological activity of* Pandanus odoratissimus* is shown in Table 7.

### 11.1. Acute Oral Toxicity Study

#### 11.1.1. In Mice

Selected animals both female and male mice administered with the extract at a dose of 2000 mg/kg showed no toxicity during the experimentation period. In both sexes of mice, body weight gain of treatment rats was not changed significantly relative to that of control. While conducting the toxicity studies, animals were observed continuously for any general behavioral changes. A significant reduction in spontaneous locomotors motility, drowsiness, and remarkably quiet behaviors were observed. Thus, the extract of* P. odoratissimus* with an LD_50_ > 2000 mg/kg is considered nontoxic through acute exposure in mice [8, 24].

#### 11.1.2. In Rats

Administration of hydroethanol leaf extract of* P. odoratissimus* Linn. 2000 mg/kg/p.o. dose did not show behavioral change (Erwin's test) at continuous observation for 4 h and intermittent of 48 h. No mortality was observed during total observation period of 14 days. LD_50_ was found to be more than 2000 mg/kg calculated by AOT425* statpgm* (Version: 1.0), Acute Oral Toxicity (OECD Test Guideline 425) Statistical Program (AOT Report) on the basis of AOT425 report 200 and 300 mg/kg doses were selected for evaluating effect as antiepileptic and for fertility effect of* P. odoratissimus* Linn. [19].

### 11.2. Antioxidant Activity

The antioxidant activity of methanolic extracts of leaves of* P. odoratissimus* by four different* in vitro* models. The lipid peroxidation was assayed by estimating the thiobarbituric acid reactive substances (TBARS) in normal rat by liver homogenates. The reduced glutathione (GSH) was assayed in liver homogenates of different concentrations of* Pandanus* methanol extract using the method of Ellman et al. The nitric oxide (NO) scavenging activity and 1,1-diphenyl, 2-picryl hydrazyl (DPPH) radical scavenging activity were measured using the methods of Sreejayan et al. and Shimada et al., respectively, using spectrophotometer. Vitamin E and normal saline were used as reference standard and control for all four by* in vitro* type of bioassy methods antioxidant measurement assays. The results showed significant antioxidant activity of* Pandanus* methanol extract in all four by* in vitro* type of bioassay methods used in this study and the IC_50_ (the half maximal inhibitory concentration of an inhibitor that is required for 50% inhibition of antioxidant activity) of plant extract was comparable to that of vitamin E, the reference standard compound used in this study. It is concluded that the methanolic extract of leaves of* P. odoratissimus* has significant antioxidant activity [20, 21].

### 11.3. Anti-Inflammatory Activity

The anti-inflammatory activity was estimated by carrageenan-induced acute and formalin-induced chronic paw edema models in rats. The methanolic extract of* P. odoratissimus* was given in the doses of 25, 25, and 100 mg/kg^−1^. The plant extract at the dose of 100 mg kg^−1^ showed significant anti-inflammatory activity at 3 h observation where, it caused increase in inhibition of paw edema by carrageenan-induced acute (68℅) and the formalin-induced chronic (64.2℅) paw edema models with standard drug diclofenac sodium in rats [22].

### 11.4. Acute Anti-Inflammatory Activity

Plants are widely used in the various traditional systems of medicine like Ayurveda, Siddha, and Unani for their analgesic, anti-inflammatory, and antipyretic activity.* P. odoratissimus* (kewda) has been used in rheumatic fever, rheumatism, and rheumatoid arthritis. The chemical composition of this essential oil, obtained by hydrodistillation of staminate inflorescences of kewda includes more than sixty components. The major components of the hydrodistilled kewda oil are 2-phenyl ethyl methyl ether terpene-4-ol, *α*-terpineol, 2-phenyl ethyl alcohol benzyl benzoate, and so forth. Kewda oil is traditionally used in earache, headache, arthritis, debility, giddiness, laxative, and rheumatism. Both methanolic and hydroalcoholic extracts were tested in rodent models by carrageenan-induced paw edema, albumin induced plantar edema, acetic acid induced vascular permeability, and castor induced diarrhoea. In all these animal models both extracts have shown significant anti-inflammatory activity [10].

### 11.5. Analgesic Activity

Analgesic activity of aqueous extract of* P. fascicularis* Lam. at doses (400 and 800 mg/kg) by using hot plate models, tail-flick method in rats. and the writhing model of mouse and compared with the analgesic action of codeine and aspirin.* Pandanus* aqueous extract revealed significant analgesic activity by both central (*P* < 0.001) and peripheral (*P* < 0.001) mechanisms in this study, which is comparable to that of codeine and aspirin, and this favors the use of* Pandanus* aqueous extract in rheumatism and rheumatoid arthritis in traditional medicine [23].

### 11.6. Antidiabetic Activity

The roots of* P. odoratissimus* aqueous extract were tested for its effect on blood glucose levels in normal and diabetic rats. Hypoglycemia was observed in basal condition when tested at an oral dose of 75, 150, and 300 mg/kg body weight. The ethanolic extract has displayed a significant dose-dependent antihyperglycemic activity in oral glucose tolerance test and also found to reduce the increased blood glucose in alloxan-induced diabetic rats (31℅ at 150 mg/kg and 51℅ at 300 mg/kg body weight). Chronic administration (10 days) of the ethanolic extract of extract of root significantly reduced the blood glucose in alloxan-induced diabetic rats. The extract was also found to reduce the increased blood urea and inhibit the body weight reduction and leucopenia induced by alloxan administration. The ethanolic extract was also found to effectively scavenge the DPPH and lipid peroxide free radicals by* in vitro* type of bioassay methods with an IC_50_ value of 10 and 8 *µ*g/mL, respectively. The preliminary phytochemical examination reveals the presence of flavanoids and tannins, which may be attributed to observed antioxidant and significant antihyperglycemic properties [24–26, 45].

### 11.7. Antimicrobial Activity

The antimicrobial effects of petroleum ether, chloroform, and hydroalcoholic extracts of* P. odoratissimus* leaf against* Bacillus subtilis*,* Escherichia coli*,* Staphlococcus aureus*, and* Candida albicans*. In terms of antimicrobial effects, all the three extracts exhibited effective inhibition zones against gram-positive bacteria, that is,* S. aureus, B. subtilis*. However, they were ineffective against gram-negative bacteria (*E. coli* and* P. aeruginosa*) and fungi (*C. albicans*). The minimum inhibitory concentration (MIC) of hydroalcoholic, chloroform, and petroleum ether extracts was found to be 25, 50, and 50 mg/mL, respectively, against gram-positive bacteria. Out of three extracts, hydroalcoholic extract showed good antimicrobial activity. The phytochemical study showed the presence of alkaloids and flavonoids in hydroalcoholic extract, which might be responsible for its good antimicrobial activity [29, 30].

### 11.8. Antifungal Activity

Keratophilic fungi, a type of dermatophytes, cause infection to hair, glabrous skin, and nails of human beings and animals. Soil is well known to be supporting the transient existence of them. The volatile plant oils have been of concern recently to develop a new antifungal agent. Four different commercially available Itra-Bella (*Lonicera x bella zabel*), kewda (*Pandanus odoratissimus*), Rajnigandha (*Polianthes tuberosa*), and Mogra (*Jasminum sambac*) for their antifungal activity against the* Aspergillus flavus*,* Trichophyton mentagrophytes*,* Trichophyton tonsurans*,* Trichophyton verrucosum*,* Epidermophyton floccosum*, and* Microsporum nanum* were isolated from soil.

The diameters of zone of inhibition formed by Bella, Rajnigandha, kewda, and Mogra against* T. tonsurans* were observed to be 47 mm, 34 mm, 17 mm, and 30 mm, respectively, while it was 30 mm, 22 mm, 15 mm, and 17 mm against the* M. nanum*. The activity was observed quite low against the* Aspergillus flavus* with 17 mm, 15 mm, 11 mm, and 13 mm inhibitory zone shown by Bella, Rajnigandha, kewda, and Mogra, respectively, while it was intermediate against the* Epidermophyton floccosum* with 27 mm, 20 mm, 21 mm, and 15 mm of zone inhibition. Thus, the maximum antifungal effect was shown by Bella with 47 mm inhibitory zone against the* T. tonsurans*, and the minimum was 11 mm shown by kewda against the* Aspergillus flavus*. Significantly, the antifungal activity shown by few Itra was comparatively better than control (antifungal drugs, Terbinafine, Itraconazole, and Fluconazole) [32].

### 11.9. Antiviral Activity

A lectin, designated Pandanin, was isolated from the saline extract of the leaves of* P. amaryllifolius*, using ammonium sulfate precipitation, affinity chromatography on mannose-agarose, and molecular size exclusion by gel filtration. Pandanin is an unglycosylated protein with a molecular mass of 8.0 kDa both after gel filtration and on sodium dodecyl sulfate-polyacrylamide gel electrophoresis, indicating that it is a single polypeptide chain. These first isolated 10 residues of the N-terminal amino acid sequence are DNILFSDSTL. An analysis of the sequence of first 30 amino acids at the N-terminal region shows that Pandanin has about 50–60% the quality of being similar or corresponding in position or value or structure or function (homology) to those of mannose-specific lectins reported from monocot plants. Pandanin exhibits hemagglutinating activity toward rabbit erythrocytes, and its activity could be reversed exclusively by mannose and mannan. Pandanin also possesses antiviral activities against human viruses, herpes simplex virus type-1 (HSV-1), and influenza virus (H1N1) with 3 days of EC_50_ of 2.94 and 15.63 *μ*M, respectively [33].

### 11.10. Hepatoprotective and Hepatocurative Activity

In developing countries like India, hepatic disorders are steadily increasing.* Ketaki* (*P. odoratissimus* Roxb) is an important traditional medicine used in northern Karnataka (India) for jaundice. The experimental model was adopted by Watanabe and Takita (1973) to evaluate the hepatoprotective and hepatocurative activities of a* Pandanus* root decoction on CCl_4_ induced liver damage in albino rats. The degree of protective and curative activity was determined by measuring the levels of serum glutamate oxaloacetate transaminase (SGOT), serum glutamate pyruvate transaminase (SGPT), alkaline phosphatase (ALP), total serum bilirubin, and serum albumin. Histological studies and all haematological parameters have promoted the hepatocurative activity.* Pandanus* root decoction was found to be hepatocurative but not hepatoprotective [27, 28].

### 11.11. Hepatotoxic Activity

The antioxidant effect of methanol extract of* P. odoratissimus* leaf in Wistar albino rats administered with carbon tetrachloride (CCl_4_) at 1.5/kg^−1^ CCl_4_ 1 mL/kg^−1^ in liquid paraffin 3 doses (i.p.) at 72 h interval. The extracts at the doses of 50 and 100 mg/kg^−1^ and (Liv-52) 25 mg/kg^−1^ were administered to the CCl_4_ treated rats. The effect of extract and Liv-52 on serum transaminase (GOT, GPT), alkaline phosphatase (ALP), bilirubin, and total protein were measured in the rat induced by CCl_4_. The effects of extract on lipid peroxidation (LPO), superoxide dismutase (SOD), catalase (CAT), glutathione (GSH), and vitamin E were estimated. The extract and Liv-52 produced significant (*P* < 0.05) effect by decreasing the activity of serum enzymes, bilirubin, uric acid, and lipid peroxidation and significantly (*P* < 0.05) increased in the level of SOD, CAT, GSH, vitamin E, and protein. Hence these results suggest that methanolic extract of* P. odoratissimus* has potent antioxidant properties [27].

### 11.12. Free Radicals Scavenging Activity

*Pandanus* is used in traditional use as a Ayurvedic medicines and it is also famous for its frequency. The antioxidant activity of methanolic extract of* Pandanus* was studied by its ability to scavenge DPPH, nitric acid, superoxide radicals, and hydroxyl radicals. The plant extract shows antioxidant activity by 87.52℅ reducing the DPPH and 73.55℅ inhibition of nitric acid. The result also indicates maximum inhibition of superoxide radical's inhibition 74.12℅ and 78.14℅ inhibition of hydroxyl radicals. The butylated hydroxytoluene (BHT) was used as standard antioxidant [21].

### 11.13. Antidiuretic Activity

The ethanol and aqueous extracts of* Pandanus* are claimed as an antidiuretic by some traditional practitioners. Furosemide was used as a diuretic agent to induce diuresis. Vasopressin (antidiuretic hormone; ADH) was used as a standard. The results demonstrated both the ethanol and aqueous extracts of* Pandanus* and ADH significantly impaired the total urine output. However, antidiuretic potential of ethanol extract was similar to that of ADH. The extracts caused a significant decrease in natriuresis and kaliuresis. It has the potential to impart therapeutic effect in diuretic [25].

### 11.14. Helminthic Activity

*P. odoratissimus* Linn. is found in the tribal area of Koraput district and extensively used traditionally by the tribal people as anthelmintic, rheumatism, stimulant, headache, and antispasmodic. The preliminary investigation of phytochemical constituents of ethyl acetate and ethanol extracts of leaves of plant* P. odoratissimus*. The two doses (25 and 50 mg/mL) of extracts were evaluated for their anthelmintic activities on adult Indian earthworms,* Pheretima posthuma*. The activities are comparable with the standard drugs, piperazine citrate and albendazole. All the doses of ethyl acetate and ethanol extracts of* Pandanus* showed better anthelmintic activity than the standard drug albendazole except ethyl acetate extract at 25 mg/mL of concentration. The extracts of ethyl acetate at concentration of 25 and 50 mg/mL showed lesser anthelmintic activity than the standard drug piperazine citrate. When the dose of the extract is increased, a gradual increase in anthelmintic activity was observed [34].

### 11.15. Antitumour Activity

Antitumour activity of ethanol extract* Pandanus* was evaluated against Dalton's ascitic lymphoma (DAL) tumour model on dose-dependent manner. The activity was assessed using survival time, average increase in body weight haematological parameters, and solid tumour volume. Oral administration of alcoholic* Pandanus* extract increased the survival time and decreased the average body weight of the tumour bearing mice. After 14 days of inoculation, EPF was able to reverse the changes in the haematological parameters, protein and PCV consequent to tumour inoculation. Oral administration of EPF was effective in reducing solid tumour mass development induced by DAL cells. The results showed that EPF possess significant activity in dose-dependent manner [46].

### 11.16. CNS-Depressant Action

The effect of methanolic extract of* P. odoratissimus* leaf on the CNS was studied by using different neuropharmacological paradigms including spontaneous motor activity, rota-rod performance, and potentiation of Pentobarbital sodium sleeping time in albino mice. Preliminary phytochemical evaluation and acute toxicity studies were also carried out where LD_50_ > 2000 mg/kg was considered nontoxic through acute exposure in rats by the oral route. The* Pandanus* extract (50, 100, and 200 mg/kg i.p.) produced a reduction in spontaneous motor activity, motor coordination and prolonged Pentobarbital sodium sleeping time. These observations suggest that the leaf of* P. odoratissimus* contains some active principles which possess potential CNS-depressant action [8].

### 11.17. Antiepileptic/Anticonvulsant Activity

Increase in latency to seizures as well as reduction in duration and frequency of seizures indicated anticonvulsant activity. The selected extract was more effective in all models used except the strychnine-induced convulsions.* P. odoratissimus* ethanol extract (100 and 200 mg/kg body wt.) significantly (*P* < 0.05 to 0.01) shortened the duration of convulsions in maximum electroshock and picrotoxin induced seizures. Delay in the onset of convulsions in the two tests was significant (*P* < 0.01). Reduction in the frequency of seizures was also significant (*P* < 0.05, 0.01) in both tests.* P. odoratissimus* further delayed the onset of seizures in picrotoxin induced seizures model while producing (66.7 and 83.33%) protection against death in mice [19].

## 12. Pharmaceutical Uses

### 12.1. Tannin/Dye

A black dye used in weaving is prepared from the roots in Kiribati. Charcoal from* Pandanus* was used in various mixtures to dye and waterproof canoes.

### 12.2. Cosmetic/Soap/Perfume

Male flowers picked from uncultivated* Pandanus* are used alone or in combination with other flowers to perfume coconut oil in Polynesia. An exquisite, uniquely Pacific perfume is made from the aromatic fruits of selected traditional cultivated varieties in the Cook Islands. In Southland Southeast Asia, the male flowers and preparations derived from them are used to scent clothes and incorporated into cosmetics, soaps, hair oils, and incense sticks. In Hawaii, the male flowers were used to scent tapa.

### 12.3. Pandan Edible Colouring and Flavouring Powder

A study on the production of spray-dried pandan (*P. amaryllifolius*) powder was conducted and optimized using response surface methodology (RSM). Parameters investigated include inlet temperature (170–200°C) and feed rate (6–12 rpm), with a preset outlet temperature of 90°C. The estimated regression coefficients (*R* ^2^) for the physicochemical characteristic and sensory responses of pandan powder were ≥0.800, except for overall acceptability. Some mathematical models could be developed with confidence based on the results from all responses. An optimum drying process for spray drying represents conditions that would yield acceptably high colour index (such as *L* value, *a* value, and *b* value), low moisture content, low water activity (*a*_*w*_), high solubility and high colour, flavour, odour, and overall acceptability for sensory responses. Optimum conditions of 170°C inlet temperature and 6 rpm feed rate, with a constant outlet temperature of 90°C, were established for producing spray-dried pandan powder as an edible colouring and flavouring powder [47].

### 12.4. Separation of Divalent Metal Ions

Desorption by dead biomass has been studied on* P. amaryllifolius* Roxb (*Pandanus* leaves) by conducting batch experiments. The recovery of heavy metals such as lead and copper ions from biomass was examined using a variety of desorbing chemicals. This study aims to discover the best chemical which is able to leach the metal effectively with highest desorbing capacity. The results showed that HCl at pH 2 and 3.0 mM EDTA at pH 4.58 were effective in desorbing the copper and lead ions from the biomass. The recovery of copper is very feasible since over 90% of copper was removed from the biomass. The percentage of lead recovery is about 70%. In contrast, Na_2_CO_3_ and NaOH are not effective in desorbing both of the metals. The results indicated that low PH is preferable for desorbing the metal ions. The binding ability of HCl is explained using ion-exchanging principle. More concentrated protons are able to replace those ions thus regenerating the biomass. EDTA is functioning as polydentate ligands, which appear to grasp the metal between the six donor atoms. It was suggested that recovery of metal ions is mainly due to the strength of bonding between the fraction of functional group of biomass and metal ions. Recovery of the deposited metals can be accomplished because they can be released from the saturated biomass in a concentrated wash solution, which also regenerates the biomass for reuse. Desorbing chemicals such as HCl and EDTA have proved successful for desorbing the metal ions. Thus, biosorption of heavy metals by biomass will be emerged as one of the alternative technologies in removing the heavy metals [48].

### 12.5. A Natural Cockroach Repellent Activity

Seven compounds and fractions prepared from pandan leaves (*P. amaryllifolius*) were evaluated for repellent activity against* Blattella germanica* Linn. using a modification of the linear tract olfactometer. 2-Acetyl-1-pyrroline, pandan essence, and the hexane-pandan extract were repellent (65–93% repellency) at all concentrations tested; the acetone-pandan extract was attractive at increasing concentrations (minimum of 62% attractancy); artificial pandan flavouring and the dichloromethane-pandan extract gave erratic results. Undiluted crude aqueous pandan extract displayed an attractancy of 62%. The potential of* P. amaryllifolius* as a natural and environmentally friendly pest management tool is discussed [49].

## 13. Analytical Evaluation

### 13.1. HPTLC Analysis for Polyphenols

A densitometry HPTLC analysis was performed for the development of characteristic finger printing profile. The* P. odoratissimus* methanolic extract of root was dissolved with HPLC grade methanol 100 mg/0.5 mL. The solution was centrifuged at contents which were centrifuged at 3000 rpm for 5 min and used for HPTLC analysis. Then 2 *μ*L of the samples was loaded as 7 mm band length in the 10 × 10 Silica gel 60F254 TLC plate using Hamilton syringe and CAMAG LINOMAT 5 instrument. The samples loaded plate was kept in TLC twin through developing chamber (after saturation with solvent vapor) with respective mobile phase (polyphenolic compound) and the plate was developed in the respective mobile phase (toluene-acetone-formic acid 4.5 : 4.5 : 1) up to 90 mm. The developed plate was dried using hot air to evaporate solvents from the plate and sprayed with stannic chloride reagent. The plate was kept Photo-documentation chamber (CAMAG REPROSTAR 3) and captured the images at UV 366 nm. Finally, the plate was fixed in scanner stage and scanned at 254 nm. The upsurging interest in the health benefits of the Peak table, Peak display, and Peak densitogram was identified [50].

### 13.2. High Performance Liquid Chromatography

Fifteen (1–15) compounds including ten phenolic compounds and five flavonoids were isolated from the fruits of* Pandanus tectorius*. All of the compounds were isolated and purified by various column chromatographies especially by semipreparative high performance liquid chromatography (HPLC). Their structures were determined under the aid of spectral methods. All compounds were isolated from this medicinal plant for the first time. The biological activities of some compounds were discussed according to the results of related literature [51].

## 14. Conclusion

The scientific research on* P. odoratissimus* suggests a huge biological potential of this plant. It is strongly believed that detailed information as presented in this review on the phytochemical and various biological properties of the plant might provide detailed evidence for the use of this plant in different diseases.

*Pandanus odoratissimus* is said to be restorative, indolent, promoting a feeling of well-being and acting as a counter to tropical climates. It may be chewed as a breath sweetener or used as a preservative in foods. It is also believed to have health-related properties, including antiviral, antiallergy, antiplatelet, anti-inflammatory, antioxidant, and anticancer action. Ayurvedic science has found the medicinal action of essential oil yielded by the screw pine's highly scented flowers to be useful in headaches, earaches and as a liniment for rheumatic pains. The distilled water from flowers is used for inducing perspiration.

It is also prescribed as a stimulant aphrodisiac and an antispasmodic agent. The flowers are powdered and included in medicines, which are either sniffed like snuff or smoked for asthma and other bronchial infections [52]. The leaves are thought to be useful in leprosy, smallpox, scabies, and diseases of the heart and brain.

Preliminary qualitative chemical studies indicated the presence of lignan, isoflavones, phenolic contents, steroids, saponin, terpenoids, glycosides, tannins, flavonoids, and phenolic in the extract. And isoflavones, polyphenol, namely, lignan, are responsible for regulating the rat fertility [17]; maybe it is helpful for the regulating and enhancing the human fertility [17, 19, 53].

On the basis of biological activities of* P. odoratissimus*, crude extract and derived phytochemicals and their uses as pharmacological agents in traditional and modern research are possible but will first require more clinical trials and product development. The current evidence is largely limited to correlation between identified phytochemicals and mode of action for any pharmacological activity.

Mechanisms of action studies are expected to lead the way into the discovery of new agents with improved and intriguing pharmacological properties. This could be achieved by molecular modeling studies involving interaction of bioactive phytochemicals from* P. odoratissimus* with their respective molecular and therapeutic targets. The extract of* P. odoratissimus* could be further explored in the future as a source of useful phytochemicals for the pharmaceutical industry [17].

## Figures and Tables

**Table 1:** Synonyms of *Pandanus odoratissimus*.

Botanical	*Pandanus odoratissimus *Lam. or *Pandanus fascicularis* Lamk. and *P. tectorius* [3]

Bengali	Keora, keya, and ketaki

English	Umbrella tree, screw pine, and screw tree

Gujarati	Kevda, ketak

Hawaiian	Hala (*P. tectorius*)

Hindi	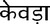 , 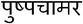 , 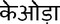 , 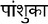
	Kewra, kewda, pushpa-chamar, keora, panshuka

Kannada	Kedige, ketake, and tale hu

Malayalam	Kaitha, kainari

Marathi	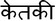 , 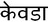 , 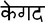
	Ketaki, kewda, kegad

Nepali	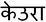 , 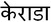 , 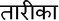 ,
	Keura, kerada, and tarika
	Kiora, keura, and kevra

Sanskrit	Ketaka

Tamil	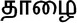 , 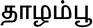 , 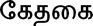
	Tazhai, talai, tazhambu, talambu, and ketakai

Telugu	Mogheli, mogil, gedaga, ketaki, and gojjangi

Urdu	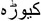 , جمبالا, جمبول, پانشکا, کیتکی
	Kiura, kevara, jambala, jambul, panshuka, and ketaki

Russian	Pandanus aromatnejshi

Japanese	Adan, takonoki

**Table 2:** Plant monograph.

Biogeography and ecology Plant name: *Pandanus odoratissimus* Linn Kingdom: Plantae-plants Subkingdom: Tracheobionta Family: Pandanaceae Genus: *Pandanus* L. F. Species: *Pandanus odoratissimus* [9, 10] *Botanical description* *Flowers* *Male flowers* A large, terminal, pendulous, compound, leafy, raceme, the leaves of which are white, linear-oblong, pointed, and concave; in the axill of each, there is a single thyrsus of simple, small racemes, of long-pointed, depending anthers; they are not sessile, but raised from the rachis of the raceme by tapering filaments. *Female flowers* A different plant, terminal and solitary, having no other calyx or corol than the termination of the three rows of leaves forming three imbricate fascicles of white floral leaves, like those of the male raceme, which stand at equal distances, round the base of the young fruit. Germs numerous, collected in firm wedge-shape angular bundles from six to ten or more (these form the compound germs of the future drupes), closely impacted round the receptacle. *Fruit* compound; oval, from five to eight inches in diameter, and from six to ten long, weighing from four to eight pounds; rough, rich orange-colour, composed of drupes numerous, wedge-shape, angular; when ripe, their large or exterior ends are detached from one another and covered with a firm, deeper orange-colored skin; apices flat, consisting of as many angular, somewhat convex, tubercles, as there are cells in the drupe, each crowned with the withered stigma, internally; the exterior half of these drupes (next the apex) consists of dry spongy cavities, their lower part next to the core or common receptacle is yellow, consisting of a rich-looking, yellow pulp, intermixed with strong fibres; here the nut is lodged. *Nut* Each drupe compound, top-shape, exceedingly hard, angular, containing as many cells as there are divisions on the apex of the drupe; each cell is perforated above and below. *Seed* Single, oblong, smooth, adhering lengthways to a small fascicle of strong, white fibers, which pass through the perforation of the cell. By far the greatest numbers of these cells are barren. It is a native of the warmer parts of Asia. All soils and situations seem to suit it equally well; it flowers chiefly during the rainy season. It grows readily from branches, whence it is rare to find the full grown ripe fruit. The male is by far the most common, a circumstance merely accidental; for I have seen some old extensive hedges entirely female, owing to their having been originally a female plant or plants nearest to these places. *Trunk* A plant may be found with a single, pretty v erect one, often feet in height, and a ramous round head; but this is seldom, for it is generally in form of a very large, ramous, spreading bush. From the stems or larger branches issue large carrot-shape, obtuse-pointed, roots, descending till they come to the ground, into which they enter and then divide. The substance of the most solid wood is something like that of a cabbage stem and by age acquires a woody hardness on the outside. *Leaves* confluent, stem-clasping, closely imbricated in three spiral rows, round the extremities of the branches, bowing; from three to five feet long, tapering to a very long fine triangular point, very smooth and glossy, margins and back armed with very fine sharp spines; those on the margins point forward, those of the back point sometimes one way, sometimes the other style. *Stigma* Single, oval, grooved lengthwise, yellow, affixed to the outside of a two-lipped umbilicus on the apex of the germ. It is the tender white leaves of the flowers (chiefly those of the male) that yield that most delightful fragrance, for which they are so universally and deservedly esteemed; and of all the perfumes that I know, it is by far the richest and most powerful. The lower yellow pulpy part of the drupe is sometimes eaten by the natives in times of scarcity and famine, and the tender white base of the leaves are also eaten raw or boiled, at such melancholy times. The taste of the pulpy part of the drupe is to me very disagreeable. The fusiform roots, already mentioned, are composed of tough fibers; they are so soft and spongy as to serve the natives for corks; the leaves also are composed of longitudinal, tough, useful fibers.	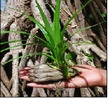 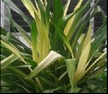 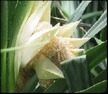 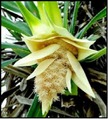 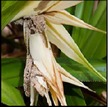 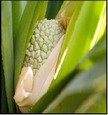 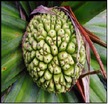 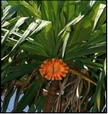 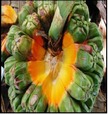 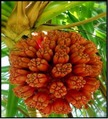 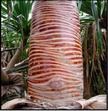
*Root* The subterranean root system is concentrated in the surface soil layers. Apart from the aerial and prop roots, the tree's root system is unlikely to interfere with maintenance or recreational activities, lawns, or structures such as sidewalks or foundations [1].	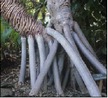

**Table 3:** Phytochemical extracts and their characteristics.

Extracts	% dry wt. in g.	Colour	Odour	Consistency
Alcoholic	11.48	Blackish green	Own characteristic	Sticky

Successive extraction				
Petroleum Ether (40–60°C)	2.08	Dark brown	Own characteristic	Waxy
Chloroform	2.66	Dark Green	Own characteristic	Powder
Ethyl acetate	1.91	Brownish yellow	Own characteristic	Sticky
n-Butanol	2.11	Brown	Own characteristic	Sticky
Methanol	8.24	Reddish Brown	Own characteristic	Sticky

**Table 4:** Phytochemical structures in *Pandanus*.

Chemical name	Chemical structures
Norpandamarilactonine-A	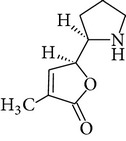

Pandamarilacton-32	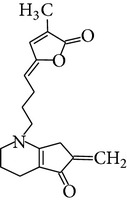

Norpandamarilactonine-A	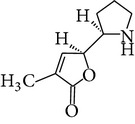

Norpandamarilactonine-B	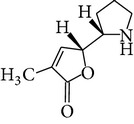

Pandamarilactone-1 (C_18_H_25_NO_4_)	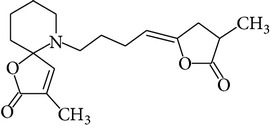

Pandamarilactonine-A (C_18_H_25_NO_4_)	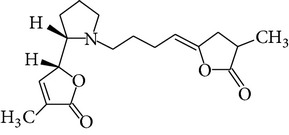

Pandanamine (C_18_H_23_NO_4_)	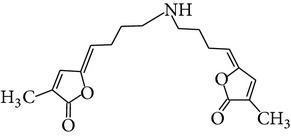

Pandamarilactonine-C, -D	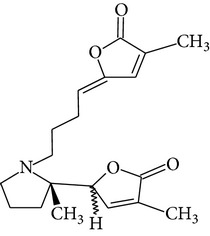

Norpandamarilactonine-A	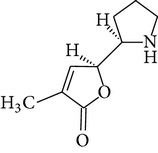

Pandanamine	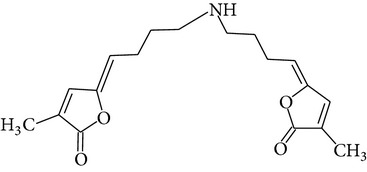

Pandamarilactone-31	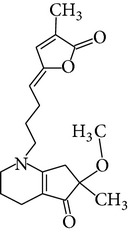

Artifact	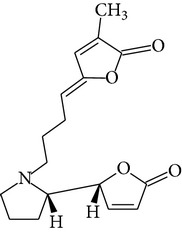

A compound which is a not “Natural product” (C_18_H_23_NO_4_)	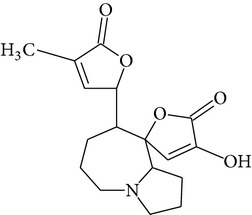

Pandamarine	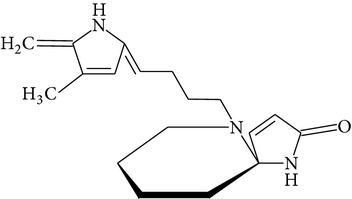

Pandamarilactam-3y	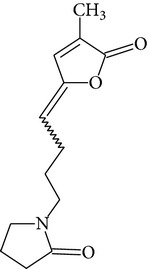

Ascorbic acid (vitamin C)	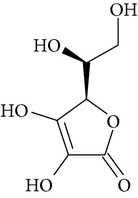

Riboflavin (vitamin B_2_)	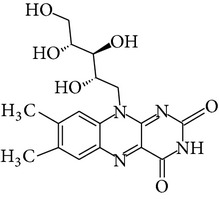

Thiamine (vitamin B_1_)	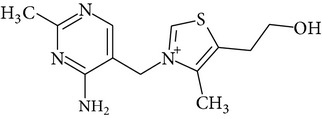

Nicotinic acid/niacin (vitamin B_3_)	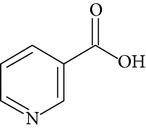

**Table 5:** Nutritional aspects of *Pandanus* or screw pines.

	Edible pericarp^*^	*Pandanus* paste^#^	References
Per 100 g of it Contents of pandan	228 kilocalories,	321 kilocalories,	([11–13])^*^ ([14–16])^#^
	water (80 g),	2.2 g protein,	
	carbohydrates (17 g),	134 mg calcium,	
	beta-carotene (19 to 19,000 *μ*g)	108 mg phosphorus,	
	vitamin C (5 mg),	5.7 mg iron,	
	protein (1.3 mg),	0.04 mg thiamin,	
	fat (0.7 mg),	2 mg vitamin C,	
	fiber (3.5 g)	390 to 724 *μ*g beta-carotene (vitamin A)	[12, 13]

*Pandanus* pulp	Usually prepared as a drink; mixed with coconut cream makes a tasty, sweet food item		[12, 13]

Flesh of deeper yellow- and orange-colored pandan keys	Adults may consume 20–50 keys typically; highly pleasurable, 50% of energy intake		[11]
	As carotenoid (provitamin-A) rich food may protect against diabetes, heart disease, and cancer and alleviate these serious emerging problems		[11]

Fresh *Pandanus* fruit	Vitamin C (ascorbic acid), thiamine, riboflavin, and niacin (vitamin B-3)		[14, 15]
	Juice and jam In parts of Micronesia, chewing		[11, 15]

**Table 6:** Ethnomedicinal uses of *P. odoratissimus*.

Plant parts	Medicinal uses
Leaves	Leprosy, aphrodisiac, scabies, anxiety and heart disease, leucoderma, tumors, leprosy, antiepileptic, anticonvulsions, and skin disease Female rats fertility regulator [17]
Flower	Headaches, earaches, antispasmodic, and aphrodisiac [17]
Root	Antidiabetics, antidote, abortifacient; skin diseases, leprosy, scabies, and syphilis [18]
Oil	Rheumatoid arthritis, skin disease, earache, headache, arthritis, debility, depurative, giddiness, laxative, leprosy, small *Pandanus odoratissimus*, and spasms
Fruit	Vat, kaph, urinary discharge, and leprosy, male aphrodisiac [17]

**Table 7:** A summary of reported pharmacological activity of *P. odoratissimus* [17].

Species/method used	Property	Source
Wistar rats	Antiepileptic and anticonversant	[19]
	Antioxidant	[20, 21]
	Anti-inflammatory	[22]
	Acute anti-inflammatory	[10]
	Analgesic	[23]
	Antidiabetic	[24–26]
	Diuretic activity	[25]
	Hepatotoxic	[27]
	Hepatoprotective & hepatocurative activity	[27, 28]
	Fertility enhancer and regulation activity, in female rats	[17]
	Sex stimulant activity, in male rats	[17]
	Aphrodisiac	

Swiss albino mice	Antidiabetic	[24–26]
	CNS depressant activity	[8]

*Bacillus subtilis*, *Escherichia coli*, *Staphylococcus aureus*, and *Candida albicans *	Antimicrobial activity	[29–31]

*Aspergillus flavus*, *Microsporum nanum*, *Epidermophyton floccosum*, *Trichophyton tonsurans*, Mentagrophytes, and Trichophyton verrucosum	Antifungal activity	[32]

Human viruses, herpes simplex, type I (HSV-1), and influenza virus (H1N1)	Antiviral activity	[33]

Inhibition of hydroxyl radicals	Free scavenging activity	[21]

Adult Indian earthworms, *Pheretima posthuma *	Anthelmintic activity	[34]
